# Ectopic Thymoma in the Thorax With Associated Myasthenia Gravis

**DOI:** 10.7759/cureus.72146

**Published:** 2024-10-22

**Authors:** Ciara Guerin, Leona Ward, Alexander Robinson, John Kirby, Muna Sabah

**Affiliations:** 1 Histopathology, St. Vincent's University Hospital, Dublin, IRL; 2 Cardiothoracic Surgery, Mater Misericordiae University Hospital, Dublin, IRL; 3 Radiology, Mater Misericordiae University Hospital, Dublin, IRL; 4 Radiology, Connolly Hospital Blanchardstown, Dublin, IRL; 5 Histopathology, Royal College of Surgeons in Ireland, Dublin, IRL

**Keywords:** ectopic thymoma, histopathology examination, myasthaenia gravis, paraneoplastic syndromes, thoracic radiology

## Abstract

A 52-year-old man presented with a one-year history of episodic unilateral facial numbness, jaw weakness, ptosis, and hoarseness. Brain imaging was unremarkable and an 11.5cm right lower thoracic mass was discovered on chest x-ray, which was further evaluated by CT and MRI radiology. The lesion was biopsied, and histopathological examination revealed a thymoma. A diagnosis of ectopic thymoma with paraneoplastic myasthenia gravis was supported by positive anti-acetylcholine receptor and anti-titin serum antibodies. Paraneoplastic syndromes are rarely reported in association with ectopic thymoma, making this case unusual. We discuss the nuances of diagnosis, classification, and staging of this rare case, as well as the patient’s subsequent management by cardiothoracic surgery and neurology specialists.

## Introduction

Thymoma arising outside of the anterosuperior mediastinum is a very rare occurrence, accounting for only 4% of thymoma cases [1]. Ectopic thymoma has been reported to occur in the cervical region, lung, pleura, thyroid gland, pericardium, middle mediastinum, and posterior mediastinum [1,2]. The presenting features of an ectopic thymoma vary depending on where it arises. In contrast to orthotopic thymomas of the anterosuperior mediastinum, reported cases of ectopic thymoma have rarely been associated with myasthenia gravis [1,2].

We present a case of a large ectopic thymoma arising in the right lower thorax of a man who presented with unilateral facial numbness, jaw weakness, ptosis, and hoarseness. We use this case to illustrate challenges posed by the diagnosis and sub-typing of ectopic thymoma.

This article was previously presented as a meeting abstract at the 2024 European Congress of Pathology on 7th September 2024. 

## Case presentation

A 52-year-old man presented with a one-year history of episodic unilateral neurological symptoms. He experienced facial numbness, jaw weakness, ptosis, and hoarseness. He did not report any respiratory symptoms, chest pain, or weight loss. He had a history of desmoid tumor excised from his lower back 17 years prior.

CT and MRI of the brain were performed, which identified no abnormalities, and routine blood tests, including inflammatory markers, were normal. His symptoms were initially attributed to a transient ischaemic attack.

**Figure 1:**
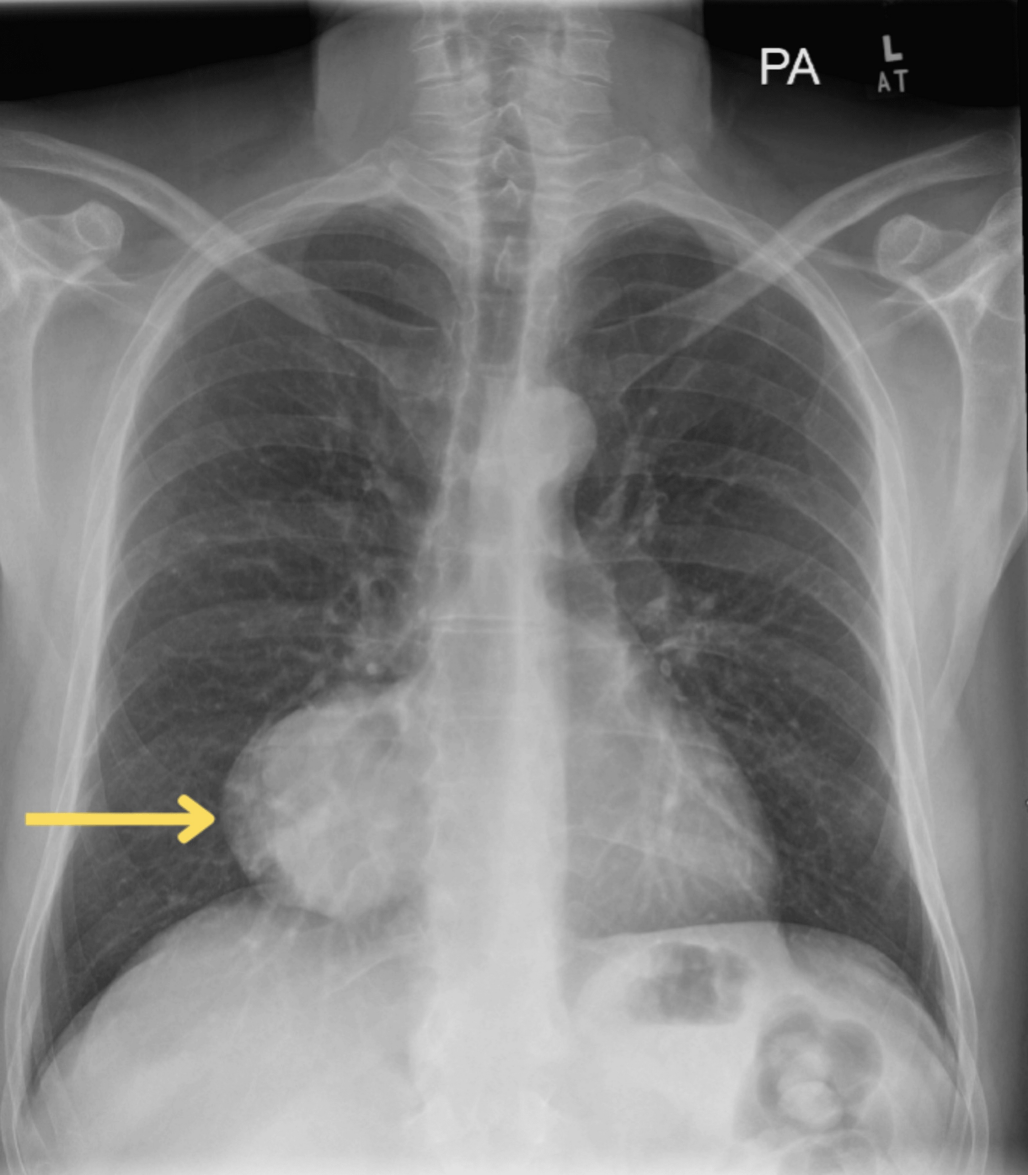
Chest X-ray showing a rounded opacity in the right lower zone, abutting the cardiac silhouette (yellow arrow)

**Figure 2:**
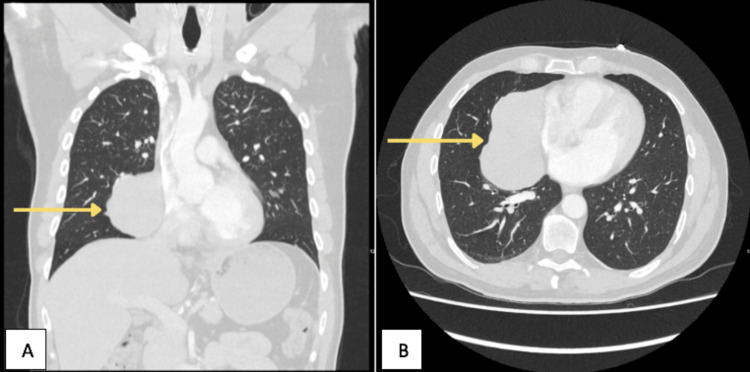
CT chest, lung window; coronal view (A) and axial view (B) showing a solid right-sided thoracic mass with a wide base abutting the pericardium (yellow arrow) and normal background lung parenchyma

**Figure 3:**
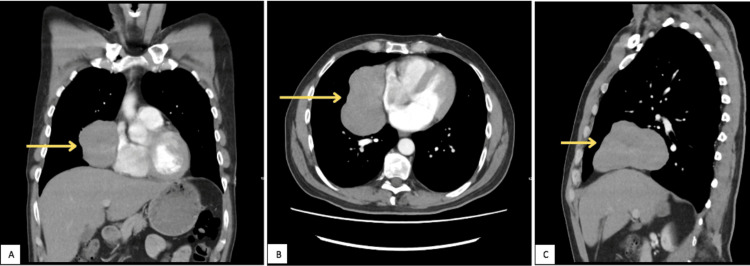
CT chest, soft tissue window; coronal view (A), axial view (B) and sagittal view (C) showing a solid right-sided thoracic mass without intralesional fat, calcification or fibrous tissue (yellow arrow)

**Figure 4:**
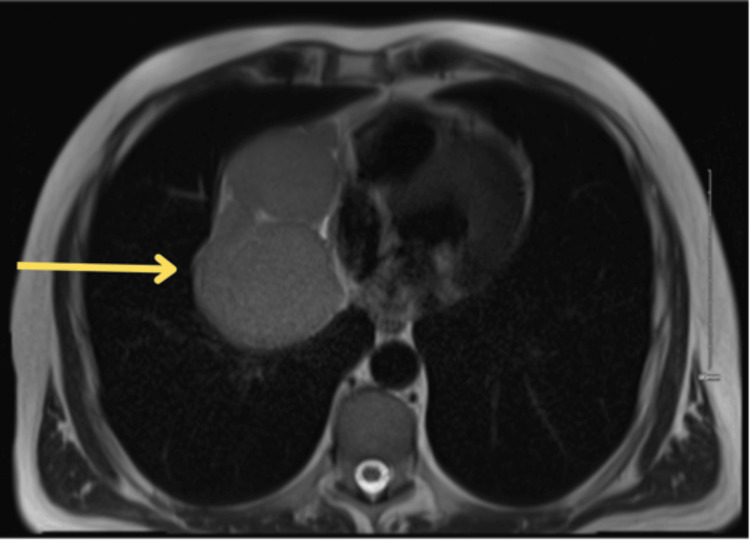
T2-weighted MRI chest pre-contrast phase showing a solid right-sided thoracic mass with internal septation (yellow arrow)

A chest X-ray (Figure 1) and subsequent CT (Figures 2, 3) and MRI (Figure 4) of the chest were performed, and collectively, they showed an 11.5cm x8.3cm x7cm solid right-sided thoracic mass with a wide base abutting the pericardium. No intralesional fat, calcification, or fibrous tissue was seen. The background lung parenchyma appeared normal, and no lymphadenopathy was detected.

A broad differential diagnosis was initially considered for the chest mass, including both benign and malignant conditions. Metastasis was felt to be unlikely, given the size of the lesion and the absence of other lesions on CT of the thorax, abdomen, and pelvis.

A CT-guided biopsy of the mass was performed. Histological examination of the tissue cores showed infiltration by hyperchromatic cells with a diffuse growth pattern. Immunohistochemical stains highlighted an epithelial population. The cells were positive for AE1/3 and p63, admixed with CD3, CD5, and TdT-positive lymphocytes. Scattered CD20-positive B-lymphocytes were present in the background. TTF-1 and CK5/6 were weakly positive in tumor cells. CD117, napsin A, CD56, chromogranin, and synaptophysin were negative. The microscopic features were in keeping with a thymoma, favored subtype B2.

Anti-acetylcholine receptor (AchR) antibody levels were raised at 373 x10-10 mol (reference range <5×10-10). Anti-titin antibodies were also found to be positive on serum analysis. The patient was diagnosed with an ectopic thymoma and paraneoplastic myasthenia gravis.

He was referred to cardiothoracic surgeons and underwent median sternotomy for resection of the thymoma. Intraoperatively, the tumor was noted to be adherent only to the superior vena cava via loose fatty tissue and was not obviously thymic in origin. It was removed en bloc using ligasure and blunt dissection. He had an uncomplicated postoperative course.

**Figure 5:**
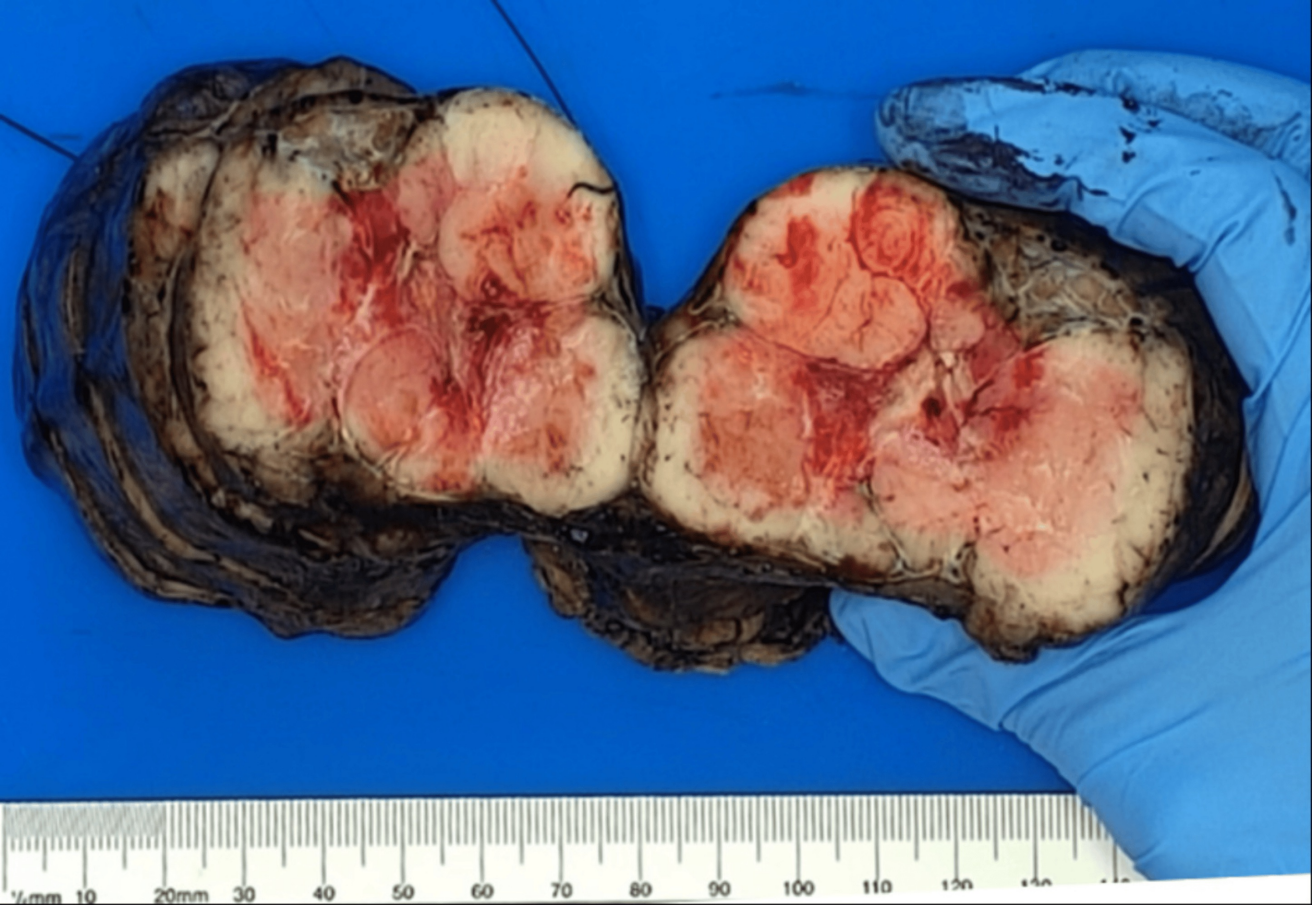
Gross features of resected thymoma

**Figure 6:**
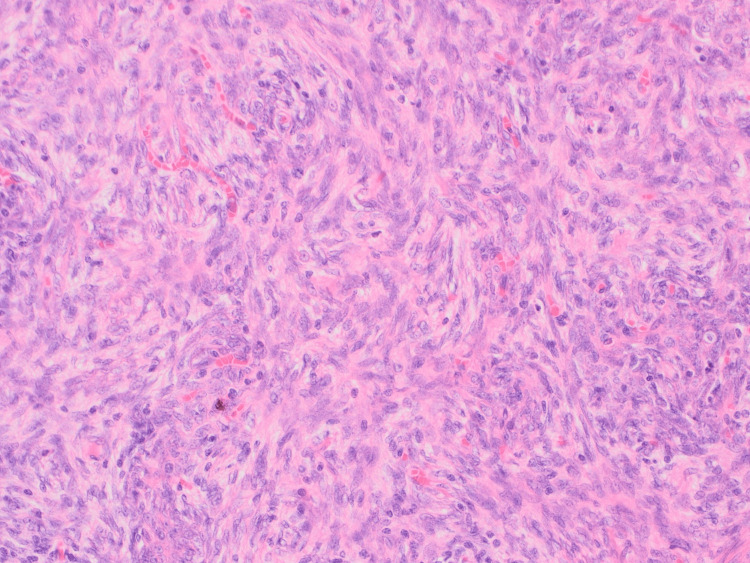
Hematoxylin and Eosin stained tumor at 20x magnification showing spindle cell/ type A component

**Figure 7:**
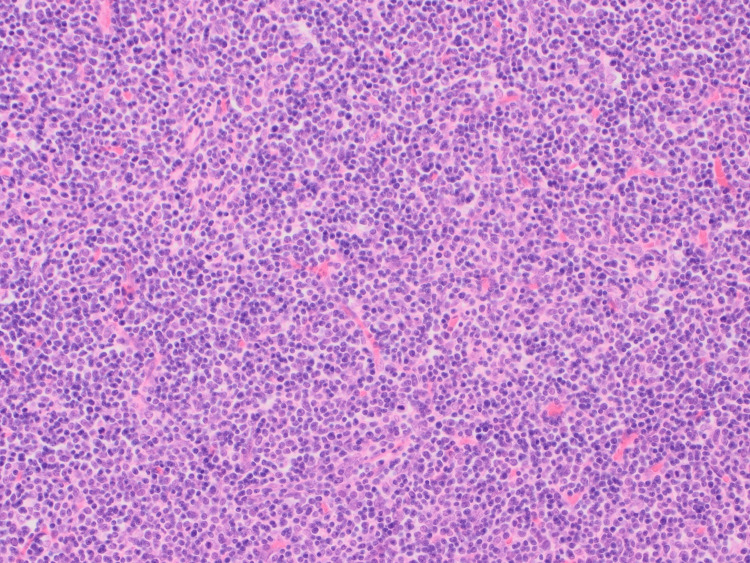
Hematoxylin and Eosin stained tumor at 20x magnification showing lymphocyte rich/ type B-like component

A gross examination of the resected tumor revealed a nodular, encapsulated mass with fibrous bands (Figure 5). Histological examination confirmed a thymic epithelial neoplasm with a lobulated growth pattern, composed of variable proportions of a spindle cell component (type A, <10%) and a lymphocyte-rich component (type B-like, >90%) with a significant population of immature T cells (Figures 6, 7). This corresponds to WHO subtype AB. The tumor was fully excised with clear surgical margins. There was no normal thymic tissue included with the specimen. The tumor cells were negative for CD117 by immunohistochemistry, a reassuring indication that the lesion was indeed a thymoma and not thymic carcinoma.

He was commenced on acetylcholinesterase inhibitor Pyridostigmine 60mg QDS preoperatively and continues this treatment with follow-up in a neurology clinic. In addition, he will be followed by cardiothoracic surgeons with repeat chest imaging every two years for the next ten years. Three months after surgery, the patient reports that his neurological symptoms have improved but not fully resolved.

## Discussion

Thymomas are neoplasms that are derived from thymic epithelial tissue. The pathogenesis of ectopic thymomas has not been fully established but two theories predominate. Ectopic thymomas are proposed either to derive from thymic tissue, which has become displaced during embryonic development, or from pluripotent stem cells in non-thymic tissues, which differentiate into thymomas [3]. Nearly 50% of ectopic thymomas develop in the cervical region, with lung and pleural locations accounting for another one-third of cases combined [1]. Less commonly, they have been reported to occur in the thyroid gland, pericardium, middle mediastinum, and posterior mediastinum [1,2].

Our patient, aged 52 years, is within the commonest age demographic presenting with thymoma. Both ectopic and orthotopic thymomas are reported to present in the 5th-6th decades [1]. Clinical manifestations of ectopic thymomas vary depending on tumor location, and as with orthotopic thymomas, they are commonly an incidental finding.

Paraneoplastic myasthenia gravis occurs in association with 30-50% of orthotopic thymomas but has rarely been reported in cases of ectopic thymoma [1,2]. The antibody, well-established as the cause of this autoimmune condition, is an anti-AchR antibody. In addition to anti-AchR antibodies, over 95% of patients with thymoma-associated myasthenia gravis also express highly specific anti-titin antibodies, which are almost never detected in myasthenia gravis without thymoma [4]. Both antibodies were found to be positive in our patient.

Post-thymectomy exacerbation of myasthenic symptoms and risk of life-threatening myasthenic crisis are recognized complications of surgery. Prophylactic plasmapheresis or intravenous immunoglobulin (IVIG) may be considered to remove circulating pathogenic antibodies [4], although they were not used in our case. Short-term use of plasmapheresis or IVIG should be considered in patients with a prior history of myasthenic crisis, low vital capacity on pulmonary function tests, or significant bulbar symptoms [5]. Our patient did not have these risk factors and myasthenic crisis does not typically occur in well-controlled myasthenia gravis. In at-risk patients, the use of these therapies reduces the risk of crisis and shortens ICU stay [5]. The choice between IVIg and plasmapheresis may be guided by the availability of these therapies and by patient factors. Plasmapheresis carries a greater risk of hemodynamic and venous access complications, whereas IVIG is contraindicated in hypercoagulable states, renal failure, and hypersensitivity to immunoglobulins [6].

The histologic features of orthotopic and ectopic thymomas are identical; however, they remain susceptible to misdiagnosis if a thymoma is not considered within the differential diagnosis of a mass outside of the anterosuperior mediastinum. Thymomas are composed of epithelial cells admixed with non-neoplastic immature T lymphocytes [1]. On a biopsy specimen, it can be challenging to identify the epithelial population amongst abundant T lymphocytes [2]. Immunohistochemical stains for epithelial and lymphoid markers aid in highlighting the two distinct cell populations. Adding to the diagnostic challenge are the varied proportions of epithelial and lymphocytic components within a thymoma and the varied morphology of epithelial cells, ranging from epithelioid to spindled cells [7]. Classification of WHO subtypes is based on these variations, and there are six possible subtypes (A, AB, B1, B2, B3, and thymic carcinoma) [7]. Subtyping requires a comprehensive assessment of the resected specimen, as tumor composition and morphology can be heterogeneous. In our case, the thymoma was of the B2 subtype based on the biopsy material, demonstrating this common pitfall. On biopsy alone, the diagnosis of ectopic thymoma may even be missed completely and may be misinterpreted as lymphoblastic lymphoma, large cell lymphoma, Burkitt lymphoma, Hodgkin's lymphoma, neuroendocrine tumours, solitary fibrous tumors, and other mesenchymal neoplasms [8,9]. In our case, clinical correlation with the history of neurological deficit raised suspicion for thymoma-associated myasthenia gravis at the time of biopsy.

The majority of thymomas, types A, AB, B1, and B2, are considered very low-grade malignant neoplasms with generally indolent behavior. Although rarely observed, thymomas of all subtypes are believed to have metastatic potential [7,9]. Type B3 thymomas, which are composed of sheets of epithelioid cells and scant lymphocytes, exhibit more aggressive behavior, such as invasion and early recurrence. Thymic carcinoma is composed of poorly differentiated thymic epithelial cells, usually manifesting as squamous cell carcinoma. KIT mutations are implicated in the pathogenesis of thymic carcinoma, and as such, CD117 positivity is found in approximately 80% of cases of thymic carcinoma and not found in other subtypes of thymoma [10,11].

Thymomas are staged according to the Tumor, Node, Metastasis (TNM) staging system. The previously popular Masaoka-Koga system, which was introduced in 1981 and modified in 1994, differentiates tumors by the degree of local extension and transcapsular invasion [12]. These two staging systems are frequently used to stage orthotopic thymomas, but staging criteria have not been established for ectopic thymomas [13].

A recognized poor prognostic factor in cases of thymoma is increased tumor size. There can be a wide size range seen at presentation, usually 4cm -15cm [8]. Larger size has repeatedly been shown to be associated with a higher risk of recurrence and metastases, with cut-off sizes differing between studies [14,15]. In the forthcoming ninth TNM classification, tumor size >5cm is proposed to be incorporated into the staging of T1 thymomas [16].

The optimum management strategy for thymoma is complete surgical resection. This is associated with the highest chance of recurrence-free survival. Incompletely resected lesions are often treated with adjuvant radiotherapy and sometimes chemoradiotherapy [17]. Close follow-up over a long time period is usual, as recurrence can occur many years after resection.

Thymectomy alone will not alleviate thymoma-associated myasthenia gravis [4], and patients require pharmacological management much the same as non-thymoma-associated myasthenia gravis. First-line therapy with anticholinesterase inhibitors is standard, and immunosuppressive drugs may be required if this does not control symptoms.

## Conclusions

Ectopic thymoma is a rare entity that can prove a challenging diagnosis. The morphological findings are the same as those of orthotopic mediastinal thymoma, and all subtypes of thymoma can develop ectopically. In contrast to mediastinal thymoma, ectopic thymoma is less commonly associated with myasthenia gravis, making our case unusual.

This case demonstrates that thymoma may be considered in the differential diagnosis for a suspicious mass in patients with myasthenia gravis, even if outside of the anterosuperior mediastinum. In particular, the presence of anti-titin antibodies should raise the index of suspicion as their detection is highly specific for thymoma-associated myasthenia gravis.

Pathologists should be aware of the heterogeneous nature of this neoplasm, which can make diagnosis and accurate subtyping challenging on limited biopsy material. Classification is best reserved following histopathological analysis of the resected specimen. An additional limitation in the assessment of ectopic thymoma is the lack of an established staging system. The use of the TNM staging system for orthotopic thymoma may not accurately reflect the prognosis and optimal management of ectopic lesions.

In this case of large ectopic thymoma, a multidisciplinary approach allowed for prompt diagnosis and treatment with a satisfactory clinical outcome.

## References

[1] Weissferdt A, Moran CA (2016). The spectrum of ectopic thymomas. Virchows Arch.

[2] Wu X, Guo J, Zhou X, Li Y, Huang Y, Wu L (2019). Ectopic thymoma: report of 2 cases and review of the literature. Int J Clin Exp Pathol.

[3] Zhou Q, Han L, Ke X, Zhou J (2020). Ectopic thymoma: retrospective analysis of eight cases with clinical features and computed tomography findings. Clin Imaging.

[4] Romi F (2011). Thymoma in myasthenia gravis: from diagnosis to treatment. Autoimmune Dis.

[5] Claytor B, Cho SM, Li Y (2023). Myasthenic crisis. Muscle Nerve.

[6] Sanders DB, Wolfe GI, Benatar M (2016). International consensus guidance for management of myasthenia gravis: executive summary. Neurology.

[7] Detterbeck FC, Parsons AM (2004). Thymic tumors. Ann Thorac Surg.

[8] (2024). Thymoma. https://app.expertpath.com/document/thymoma/6ab194f1-0775-4395-86b9-0015b266c5b1.

[9] Wang Z, Li H, Cao H, Zheng J (2014). Clinicopathological features of type AB thymoma with liver metastases. Int J Clin Exp Pathol.

[10] Kelly RJ (2013). Thymoma versus thymic carcinoma: differences in biology impacting treatment. J Natl Compr Canc Netw.

[11] Pan CC, Chen PC, Chiang H (2004). KIT (CD117) is frequently overexpressed in thymic carcinomas but is absent in thymomas. J Pathol.

[12] Detterbeck FC, Nicholson AG, Kondo K, Van Schil P, Moran C (2011). The Masaoka-Koga stage classification for thymic malignancies: clarification and definition of terms. J Thorac Oncol.

[13] Tassi V, Vannucci J, Ceccarelli S, Gili A, Matricardi A, Avenia N, Puma F (2019). Stage-related outcome for thymic epithelial tumours. BMC Surg.

[14] Bian D, Zhou F, Yang W (2018). Thymoma size significantly affects the survival, metastasis and effectiveness of adjuvant therapies: a population based study. Oncotarget.

[15] Roden AC, Yi ES, Jenkins SM (2015). Modified Masaoka stage and size are independent prognostic predictors in thymoma and modified Masaoka stage is superior to histopathologic classifications. J Thorac Oncol.

[16] Ruffini E, Huang J, Cilento V (2023). The International Association for the study of lung cancer thymic epithelial tumors staging project: proposal for a stage classification for the forthcoming (ninth) edition of the TNM classification of malignant tumors. J Thorac Oncol.

[17] Safieddine N, Liu G, Cuningham K (2014). Prognostic factors for cure, recurrence and long-term survival after surgical resection of thymoma. J Thorac Oncol.

